# Spatial Distribution and Health Risk Assessment of Dissolved Trace Elements in Groundwater in southern China

**DOI:** 10.1038/s41598-020-64267-y

**Published:** 2020-05-12

**Authors:** Congke Gu, Yan Zhang, Yu Peng, Peifang Leng, Nong Zhu, Yunfeng Qiao, Zhao Li, Fadong Li

**Affiliations:** 1 Key Laboratory of Ecosystem Network Observation and Modeling, Institute of Geographic Sciences and Natural Resources Research, Chinese Academy of Sciences, No. 11A, Datun Road, Chaoyang District, Beijing, 100101 P. R. China; 2 University of Chinese Academy of Sciences, No. 19A, Yuquan Road, Shijingshan District, Beijing, 100049 P. R. China; 3 Northwest University, 229 North Taibai Road, Xi’an, 710069 Shaanxi Province P. R. China

**Keywords:** Ecology, Environmental sciences

## Abstract

To understand the groundwater environmental quality and the impact of trace elements in the construction of urban agglomeration in China, this study collected 58 groundwater samples from the core area of the Chang-Zhu-Tan urban agglomeration (Changsha, Zhuzhou, Xiangtan) and quantitatively analyzed the content of 13 dissolved trace element and their spatial distribution characteristics. The health risk assessment model was further used to evaluate the human health risk caused by trace element pollution in groundwater. It was observed that Ba had the highest average concentration (0.28 mg·L^−1^), whereas Cd had the lowest (2.1 × 10^−5^ mg·L^−1^). Compared with China’s groundwater environmental quality standard, the exceeding rates of Se, Mn, Zn, and Ni concentrations were 37.93%, 17.24%, 1.72% and 1.72%, respectively. Ba, Cd, Co, Cr, Cu, Fe, Mo, and Pb did not exceed the corresponding standards. The 13 trace elements were distributed in a scattered pattern in space and the trace elements in both banks of the Xiang River, Zhuzhou, Weishui River and surrounding areas were relatively high. Health risk assessments showed that the carcinogenic risk values of Cd, Cr, and Pb and the health risk values of 10 non-carcinogenic elements were less than the corresponding maximum acceptable risk level. The health risks associated with non-carcinogenic substances through ingestion were higher than those associated with dermal absorption. Among the non-carcinogenic substances, Ba and Mn posed the greatest health risks. With respect to drinking water exposure, Cr had the highest carcinogenic risk, followed by Pb. Furthermore, Cd had the lowest carcinogenic risk. This study recommended that continuous monitoring of Ba, Mn, and Cr in groundwater should be practiced by assessing the risk of these elements in the Chang-Zhu-Tan urban agglomeration.

## Introduction

Groundwater is not only an indispensable element of ecological and geological environment but also a component of water resources that has a direct impact on crop growth and human life. Groundwater pollution due to dissolved trace elements has caused worldwide attention^1,2^. Although some trace elements are necessary for the normal growth of humans and animals, their excessive intake can threaten human health and animal survival due to their toxic nature for all living organisms, such as iron (Fe), zinc (Zn), copper (Cu), and manganese (Mn)^3,4^. Other potentially toxic elements, including arsenic (As), cadmium (Cd), cobalt (Co), nickel (Ni) and lead (Pb) are extremely hazardous even in minute concentrations^3^. Trace elements are hard to biodegrade and can be extracted by plants, accumulating in green plants and leafy vegetables to a very high level, which may contaminate the terrestrial food chain^5^. Intake of trace elements contaminated water is a major pathway for human exposure. Above a certain dose, most trace elements are carcinogenic and can cause serious damage to human body health^6^. Therefore, dissolved trace element pollution in groundwater can lead to a higher risk of cancer. For example, the trace metal Cd is a carcinogen with a half-life of up to 10 years, which can be retained for a long time after entering the human body^7^.

These trace elements originate from both natural (weathering and erosion of ore deposits and bed rocks) and anthropogenic (mining, industrial emission, landfill and solid waste deposits, agrochemicals and wastewater irrigation)^8–10^. With the progress of China’s urbanization, urban agglomeration is becoming the main body of future urbanization. Economic integration of urban agglomeration leads to rapid economic development; however, ecological and environmental problems become more prominent. Compared with a single city, urban agglomeration faces more complex ecological and environmental problems as well as greater threats and risks^11^. The Chang-Zhu-Tan urban agglomeration is an important economic hub in South China and an important part of the city cluster in the middle reaches of the Yangtze River. Since the 20^th^ century, the mining industry has been flourishing gradually. With the development of mineral resources, trace element pollution has become increasingly serious. Many studies have shown that fish and shrimp at the Liuyang River, which enter the Chang-Zhu-Tan urban agglomeration are unable to survive due to the discharge of industrial wastewater, which contains kerosene^12^. Enterprises on the west bank of the Xiang River directly discharge industrial wastewater into the Xiang River; some enterprises in Zhuzhou discharge release industrial wastewater into Xiang River leading to severe pollution^13–15^. Some landfills around the city cause surrounding ponds or wells to suffer pollution because of atmospheric precipitation infiltration of wastewater. Rapid development of the urban industry and agriculture leads to an increase in the amount of pollutants into the river. Domestic sewage, and untreated industrial and mining wastewater contain many trace element pollutants^16,17^. So far, researches on environmental issues related to urban agglomeration have focused on organic pollutants^18^, surface water quality grading system^11^, greenhouse gas emission^19^, soil and sediment trace element pollution^8,14,20^, among others. Further studies are needed to understand the status of groundwater trace element pollution in urban agglomeration construction and the threat it poses to the safety of residents in the core areas of the urban agglomeration.

Health risk assessment is done to evaluate the health risk of individuals exposed to harmful factors by estimating the probability of adverse effects on the human body^1,21^. At present, drinking water health risk assessment is mainly focused on the study of single urban and rural water bodies but the health risk of toxic pollutants in urban agglomeration construction has not been systematically studied. Based on this, the objectives of this research are: (1) to investigate the content and distribution of 13 trace elements (As, Ba, Cd, Co, Cr, Cu, Fe, Mn, Mo, Ni, Pb, Se, and Zn) in groundwater in the core area of the Chang-Zhu-Tan urban agglomeration, (2) to assess the potential human health risks of groundwater in the core area of the Chang-Zhu-Tan urban agglomeration. The aim is to provide a scientific basis for protecting human health and strengthening the environmental protection of water resources in urban agglomeration construction.

## Methods and materials

### Study area description

**Figure 1:**
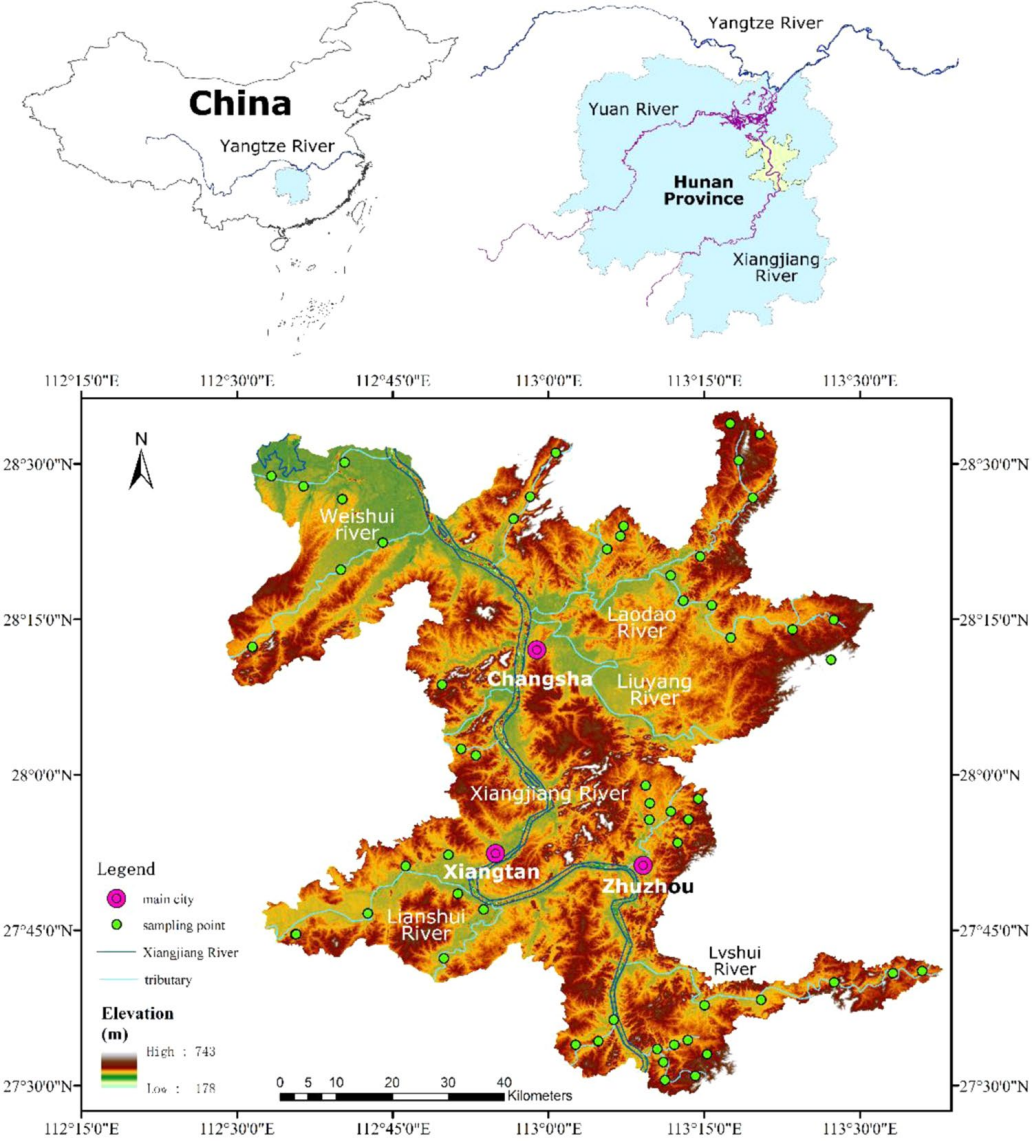
Distribution of sampling sites. (ArcGIS Desktop. 10.3. ESRI, California, US. https://desktop.arcgis.com).

The study area is the core area of Chang-Zhu-Tan urban agglomeration in Hunan province (Fig. 1). Its geographical coordinates are between 112°36′~113°16′E and 27°36′~28°33′N, and it is located in the south of the middle reaches of the Yangtze River. This research area includes three big cities: Changsha, Zhuzhou, and Xiangtan. It is in the transition zone between the hilly basin in central Hunan to the Dongting Lake Plain. The topography is high in the northeast and low in the southwest with various geomorphologic types.

The Chang-Zhu-Tan urban agglomeration is a subtropical monsoon humid climate, with an average annual rainfall of 1414 mm. The parent material of soil in the study area is complex, including plate shale, granite, sand (gravel) rock, limestone, purple rock weathering material, quaternary red clay and river, and lake alluvial material. The soil in the study area is mainly red soil and paddy soil, accounting for about 95% of the total soil area. The others include yellow soil, yellow-brown soil, vegetable soil, tidal soil, mountain meadow soil, lime soil, and purple soil.

Most of the river network water systems in the core area of the Chang-Zhu-Tan urban agglomeration belong to the Xiang River system. The Xiang River is a tributary of the Yangtze River, which is the largest river in Hunan province. In the research area, the first-level tributaries of the Xiang River are Liuyang River, Laodao River, Jinjiang River, Weishui River, Lianshui River, Juanshui River, and Lushui River. There are plenty of waters in the research area and the runoff is mainly from precipitation. The annual flow of Xiang River is 2050 m^3^/s and the annual runoff from March to July accounts for two-thirds of the annual flow.

Only 33.3% of the rural population of Hunan Province consume purified tap water as drinking water, whereas the rest of the rural population uses well water or spring water. Furthermore, 21.4% of the rural population get water for daily consumption from unprotected non-center water supply^22^. Therefore, we focused on accessing the health risk of those water sources in the countryside of study area.

### Sampling and analytical methods

The Chang-Zhu-Tan urban agglomeration is mainly built along the Xiang River and the agricultural area is concentrated along the tributaries on the east and west sides of the Xiang River. In November 2017, 58 groundwater samples labeled as G1-G58 were collected from wells near six tributaries in the core area (Fig. 1). All these chosen wells were used as drinking water source by local residents and the depth was within the range of 10–20 m. Groundwater was directly pumped from these wells. Furthermore, water that was pumped out in the first 3–5 mins was discarded for cleaning the pumping system. Then, five parallel samples were collected from each chosen well. All samples were immediately filtered through acid-treated Millipore filters (0.45 μm mesh, disposable not reusable) into pre-cleaned polyethylene-terephthalate (PET) bottles. The filtered samples were acidified to pH < 2 with ultra-purified 6 mol/L HNO_3_, preserved as about 4 °C, and then analyzed upon return to the laboratory^23^. The concentrations of dissolved trace elements (As, Ba, Cd, Co, Cr, Cu, Fe, Mn, Mo, Ni, Pb, Se, and Zn) were determined by the Inductively Coupled Plasma-Optical Emission Spectrometer (ICP-OES, PerkinElmer Co. Ltd., USA). Calibration curves were produced using quality control standards and were applied to evaluate data from each set of samples. Reagents, procedural blanks, and samples were measured six times in parallel^23^ and the average of the last three values was adopted because the first three were used to clean the pipe to avoid contamination caused by last sample. Relative standard deviation (RSD) of the three results were calculated and were found to be less than 5% for all samples of all the elements analyzed.

### Data treatment

Descriptive statistics were conducted, and concentration results of samples were compared with China’s drinking water quality standard (GB5749–2006) and the 2017 WHO Guidelines for Drinking Water Quality: First Addendum to the Fourth Edition.

Pearson correlation between interest elements was calculated to decide that if there is any significant association, indicating strong homology between them^24^. Based on the correlation coefficients, we conducted principal component analysis (PCA), a widely used statistical method to extract essential information from multidimensional data^25^, to further demonstrate the relation between elements and between groundwater samples. A varimax rotation was applied with PCA to optimize the loadings of trace elements on each principal component.

The spatial distribution of the 13 trace elements was also analyzed by the geostatistical method. According to the topography of the study area, the polluted groundwater in upstream of tributaries is not only a threat to local people, but it also poses a potential health risk to the people who live in downstream cities along the Xiangjiang River. Therefore, we picked out several elements with a high over-standard rate to exam the spatial relationship of high concentration points against a high population density area. ArcGIS 10.3 was used to make the distribution map and spatial interpolation of the experimental data, SPSS 24.0 was used for descriptive statistical analysis. Origin 2017 (OriginLab Corp., Northampton, Massachusetts, USA) was used to present loading and score plots from PCA results.

### Risk assessment index

Trace elements in groundwater enter the human body via ingestion and dermal absorption. Considering these two routes, non-carcinogenic and carcinogenic risks of corresponding elements were evaluated by a risk assessment model. All the 13 elements involved in this study have non-carcinogenic risks, while As, Cr, Cd, and Pb have carcinogenic risks. Non-carcinogenic and carcinogenic health risk assessment was determined by the Hazard Quotient (HQ) and Cancer Risk (CR). HQ and CR were calculated using Eqs. (1)–(8) adapted from the US Environmental Protection Agency^26^:

1 $$AD{D}_{ingestion}=\frac{C\times IR\times EF\times ED}{BW\times AT}$$

2 $$AD{D}_{dermal}=\frac{C\times SA\times {K}_{p}\times ABS\times ET\times EF\times ED\times CF}{BW\times AT}$$

3 $$H{Q}_{dermal}=\frac{AD{D}_{dermal}}{Rf{D}_{dermal}}$$

4 $$H{Q}_{ingestion}=\frac{AD{D}_{ingestion}}{Rf{D}_{ingestion}}$$

5 $$HQ=H{Q}_{ingestion}+H{Q}_{dermal}$$

6 $$HI=\sum HQ$$

7 $$CR=ADD\times SF$$

8 $$TCR=\sum CR$$

Where, ADD is the exposure dose contacted through ingestion of water (ADD_ingestion_) and dermal absorption (ADD_dermal_), mg kg^−1^ day^−1^; C is the average concentration of trace element in water, mg/L; IR is the drinking water ingestion rate, which is assumed to be 2 L day^−1^ for this study; EF is the exposure frequency, which is assumed as 365 day year^−1^ for this study; ED is the exposure duration, which is 74.7 years for this study; BW is the average body weight, that is 57.9 kg; AT is the averaging time for non-carcinogens and carcinogens, that is 27266 days; SA is the exposed skin area, which was 15,700 cm^2^ for this study; K_p_ is Dermal Permeability Constant, cm h^−1^, 0.0027 for As^27^; 0.002 for Cr; 0.0004 for Co; 0.0002 for Ni; 0.0006 for Zn; 0.001 for Ba, Cu, Fe, Mn, Mo, Ni, Pb, and Se^28^; ABS is the dermal absorption factor, 0.001; ET is the exposure time, that is selected as 0.2 h day^−1^ for this study; CF is the unit conversion factor, for water: 1 L = 1,000 cm^−3^; RfD is the reference dose for different analyses, expressed in mg/kg/day, which is based on U.S. risk-based assessments^26,28^. SF is the carcinogenic slope factor for ingestion, μg g^−1^ day^−1^. SF values are not available for all the selected elements, thus CR of As, Cd, Cr, and Pb are calculated only to indicate the lifetime carcinogenic risk to the local population. HI is a hazard index that indicates the aggregate risk or the risk of mixed trace elements. If HI value is less than 1, it is believed that there is no significant risk of non-carcinogenic effects. If HI value exceeds 1, then there is a chance that non-carcinogenic effects may occur, with a probability which tends to increase as the value increases^29^. TCR is the cumulative cancer risk of all carcinogenic trace elements. The acceptable or tolerable risk for regulatory purposes of TCR is within the range of 10^−6^ to 10^−4^^ 29^.

## Results and discussion

### Contents and mathematical statistics of trace elements in groundwater

The statistical results of the concentration of 13 trace elements in the 58 groundwater samples are shown in Table 1. The average concentrations indicate that Ba, Mn, Zn, Fe and As are the dominant elements in the 13 trace elements with 0.28, 0.077, 0.072, 0.017 and 0.027 mg/L, respectively. In contrast, the mean of Se, Cr, Pb, and Ni are rather low, which is around 1.3–8.5 μg/L. The average concentrations of Mo, Cu and Co are much lower than others, at 0.40, 0.21 and 0.12 µg/L, respectively. The minimum values of other trace elements, except for Ba, Mn, Zn, and Fe are all below the detection limit. Among the 13 trace elements in samples, As is the only one for which the average concentration exceeded WHO drinking water quality standard, that is 0.01 mg/L. While China’s drinking water quality standard GB5749–2006 sets the same upper-limit for As, it allows the tolerable value up to 0.05 mg/L for drinking water that was directly provided from non-center water supply. However, the concentrations of some trace elements in certain samples exceed China (GB5749–2006) and/or WHO standard^30^. According to the WHO drinking water quality standard, As is the main concern in study area as its over-standard concentration is found in 37.9% of the samples. The concentrations of Se in about 36.2% samples are higher than that of China’s standard, though they are all within the WHO standard. Cr, Mn, Ni, Pb, and Zn in several samples also exceed China drinking water quality standard. The concentrations of Cd in all groundwater samples were lower than the detection limit of our ICP-OES, despite many studies pointing out that Cd was the main pollutant in the Chang-Zhu-Tan urban agglomeration^4^. Due to the relatively high over-standard rates (showed in Table 1) of As, Se and Mn, their risk to human health in the study area are of concern.

**Table 1:** Conventional statistics of dissolved trace elements in groundwater.

Elements	Min. (mg/L)	Max. (mg/L)	Mean (mg/L)	Coefficient of variation (%)	Chinese drinking water standard^b^ (mg/L)	WHO guidelines for drinking-water quality^c^ (mg/L)	Over-standard rate (%) (compare with China standard)
As	<dl^a^	0.11	0.027	123.8	0.05	0.01	32.8
Ba	0.04	0.51	0.28	28.0	0.7	1.3	0
Cd	<dl	<dl	<0.6 μg/L	—	0.005	3.0 × 10^−3^	0
Co	<dl	5.0 × 10^−3^	<1 μg/L	100.3	0.05	—	0
Cr	<dl	7.6 × 10^−3^	3.2 × 10^−3^	46.7	0.05	0.05	0
Cu	<dl	5.4 × 10^−3^	<1.5 μg/L	83.1	1.0	2	0
Fe	7.0 × 10^−3^	0.10	0.017	84.2	0.5	0.3	0
Mn	<dl	0.70	0.077	225.1	0.3	0.4	12.1
Mo	<dl	2.9 × 10^−3^	<0.8 μg/L	78.0	0.07	0.07	0
Ni	<dl	0.05	1.6 × 10^−3^	411.2	0.02	7.0 × 10^−2^	1.72
Pb	<dl	0.01	2.6 × 10^−3^	93.1	0.01	1.0 × 10^−2^	1.72
Se	<dl	0.03	8.6 × 10^−3^	91.6	0.01	4.0 × 10^−2^	37.93
Zn	1.4×10^−3^	1.04	0.072	238.1	1.0	4^d^	1.72

^a^<dl, less than the detection line limit for As (2 μg/L), Ba (0.5 μg/L), Cd (0.6 μg/L), Co (1 μg/L), Cr (0.9 μg/L), Cu (1.5 μg/L), Fe (0.5 μg/L), Mn (1 μg/L), Mo (0.8 μg/L), Ni (0.9 μg/L), Pb (1.5 μg/L), Se (1.4 μg/L), Zn (1.2 μg/L); ^b^Chinese drinking water standard (GB5749–2006); ^c^fourth edition incorporation the first addendum; ^d^upper-limit concentration for not imparting noticeable taste in drinking water.

In terms of coefficient of variation (CV), the CV of various trace element mass concentration (As, Co, Mn, Ni, and Zn) in groundwater is more than 100%, and the variation coefficient of the trace element determination method itself is less than 10%, indicating that the trace element content in each sampling point is quite different. High CV often indicates the potential pollution by human activities. The variation coefficients of Ni, Zn, and Mn in the study area are 411.2%, 238.1%, and 225.1%, respectively, which were classified as strong variation. This was consistent with the fact that there are relatively rich deposits of non-ferrous mineral resources. Se and Mn concentrations were affected by human activities, including agricultural, mineral, and urban industrial processes^4^.

The result of Pearson correlation analysis of the 12 trace elements and pH are showed in Table 2. Cd is excluded in the table due to its extremely low concentrations. Ba and Cr contents were significantly positively correlated as well as that of Ni, Cu, and Co. Zn and Mo also showed a significant positive correlation. Se and Pb were significantly negatively correlated (Table 2). The correlation among other trace elements was not significant, indicating that the source and transport pathway in groundwater are different from other trace elements.

**Table 2:** Linear correlation coefficient matrix for the analyzed thirteen trace elements.

	pH	As	Ba	Co	Cr	Cu	Fe	Mn	Mo	Ni	Pb	Se	Zn
pH	1												
As	0.0501**	1											
Ba	−0.035	−0.008	1										
Co	−0.332*	−0.125	0.135	1									
Cr	0.099	0.22	0.429**	0.1	1								
Cu	−0.024	0.225	0.031	0.459**	0.112	1							
Fe	−0.095	−0.085	0.17	0.066	0.313*	0.028	1						
Mn	0.204	0.121	−0.049	0.098	0.156	−0.011	0.057	1					
Mo	−0.118	−0.276*	0.046	−0.097	0.004	−0.043	−0.008	0.124	1				
Ni	−0.315*	−0.087	0.062	0.968**	0.12	0.472**	0.099	0.133	−0.087	1			
Pb	−0.202	−0.041	−0.169	0.121	−0.098	0.095	0.063	−0.14	0.041	0.117	1		
Se	0.012	−0.147	0.043	−0.156	−0.146	−0.137	−0.069	−0.145	0.123	−0.164	0.420**	1	
Zn	0.035	0.116	−0.106	0.079	0.052	0.145	0.148	0.197	0.469**	0.103	−0.07	0.143	1

**Correlation is significant at the 0.01 level (2-tailed).

*Correlation is significant at the 0.05 level (2-tailed).

**Figure 2:**
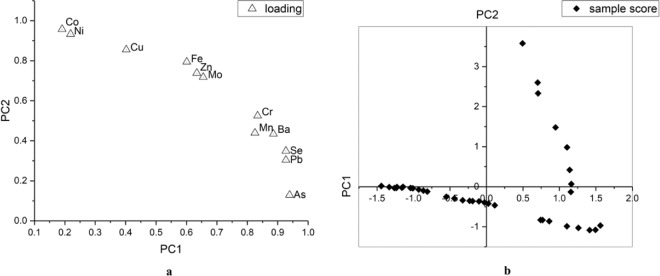
Graphical display of (**a**) the loadings of trace elements on PC1 and PC2 and (**b**) the scores of samples on PC1 and PC2.

The results of PCA with a varimax rotation were presented in Fig. 2. The eigenvalues of the first two extracted factors are higher than 1, explaining about 94.1% of the cumulative variance contribution rate. Principal component 1 (PC1) was mainly loaded with As, Pb, and Se, while the principal component 2 (PC2) strongly correlated to Co and Ni. Cd was excluded from PCA due to the concentrations of Cd in all samples were below the detection limit of our ICP-OES.

The loading plot (Fig. 2a) confirmed the results of the correlation analysis. There are relatively strong correlations between Co and Ni, Zn and Mo, Se and Pb. PC1 was dominated by As, Pb, and Se which might indicate that natural source has a significant influence on PC1 due to relative high background value of As in the soil of the study area^31^. Similarly, Mn and Ba, relatively enriched in the soil of study area, had high loading on PC1. Quite high loadings of Co, Ni, and Cu on PC2 could be explained by the strong association of these three elements in ores. Due to the prosperous metallurgical industry in cities Zhuzhou and Xiangtan, a significant amount of Co, Ni, and Cu in ores was released into the environment. Therefore, we defined PC2 as an anthropogenic source.

Figure 2b is the plot of scores of all samples on each principal component. According to the plot, most points located around the X-axis, which indicated that most samples had a relatively low absolute value of score on PC2. Only few points had high score on PC2. This result might prove that the groundwater from most sampling sites largely remained natural statues represented by PC1, while only a few sites were polluted by human activates.

### Spatial distribution characteristics of trace elements

Compared to the national standard, the over-standard rates of Se, As, and Mn in the samples are 37.93%, 32.8%, and 12.1%, respectively.

**Figure 3:**
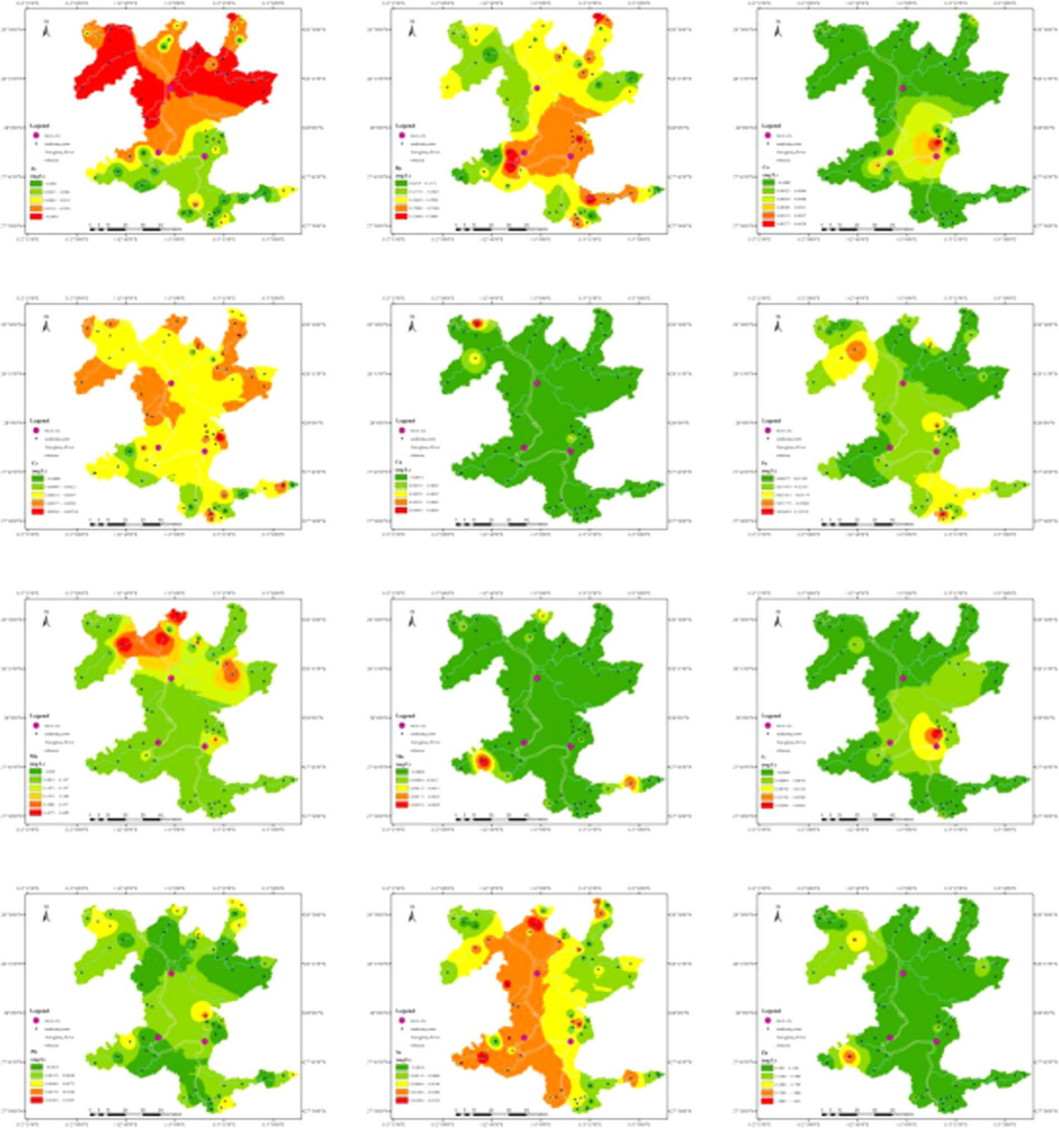
Distribution of the trace element contents in groundwater. (ArcGIS Desktop. 10.3. ESRI, California, US. https://desktop.arcgis.com).

It can be seen from Fig. 3 that the maximum concentration is three times higher than the upper limit of the national standard for drinking water (0.01 mg L^−1^). From the perspective of spatial distribution, Se content of groundwater on both sides of the Xiangjiang River exceeds the national standard. Also, regions with high Mn content in this research area are mainly distributed in the upper reaches of the Xiangjiang River. The variation coefficient of Mn (227.09%) is quite large, which is a high-intensity variation. The Xiangjiang River basin is an economically developed and urbanized area in Hunan province. Within the basin, the mining and metallurgical industries are relatively well-developed. Zhuzhou Metallurgical Corporation, Shuikoushan Mining Bureau, and Xiangtan Iron and Steel Group are distributed along both sides of the Xiang River^32^. These developed industries cause pollution of trace elements, that is Mn and Se in rivers. At the same time, the production and domestic sewage polluted by the upstream point source enters the groundwater^15^, which enhances the ion exchange between soil Mn and some components in the groundwater as well as increases the content of Mn in the groundwater.

The As in groundwater, as shown in Fig. 3, had similar tendencies as those of areas with high concentration area of C(As) > 0.01 mg/L in the north of Xiangtan and Zhuzhou city. Areas with As concentration higher than 0.05 mg/L were mainly along the Laodao River and Weishui River. The average background value of As in the Chang-Zhu-Tan Urban agglomeration soil is higher than China’s average level, with 7–16 mg/kg for paddy soil and 10–22 mg/kg for red soil. This may cause the As in soil to leach to the water in vadose zone and the As in bed rock to transfer into the groundwater in aquifer. The even distribution of As in the north of the study area also implied that the natural source was the dominant source.

China’s standard value of Zn in groundwater is 1 mg·L^−1^. Based on the spatial distribution characteristics of Zn, it was observed that Zn exceeds the limit at G48, which is located in Tuoxia village, Shitan town, Xiangtan city, Hunan province. In the sampling survey, it was found that there was no industrial source around. With the adjustment of the agricultural industrial structure, the scale of animal husbandry has been gradually developed, and the number of livestock and poultry breeding has increased exponentially. In some areas, a certain scale of animal husbandry has been formed. Zn is widely used as a feed additive in large-scale livestock and poultry breeding, which leads to a large amount of Zn in livestock and poultry excrement. When livestock and poultry excrement is applied as organic fertilizer to the soil, plants cannot fully absorb Zn, leading to Zn enrichment in soil. In the process of rainfall erosion, a large amount of sediment can enter the waterbody^33^ and Zn will adsorb and migrate together in the sediment^34^. Moreover, the interaction between surface water and groundwater will lead to Zn pollution in groundwater. In this study, ingestion is the main way to increase the Zn content in humans. The risk value of Zn through ingestion is nearly 100 times that of dermal absorption, suggesting that the intake of water is more likely to lead to toxic levels than dermal absorption.

The spatial distribution of Ni, Cu, and Co are similar, with the highest content mainly concentrated in the Longtoupu Xinglongshan Village, Zhuzhou City, Hunan Province. The Xinglongshan village has a key dispatching industrial park in Hunan province - Xinglong Industrial Park. Zhuzhou Tianlong Chemical Industrial Company, together with the Tianli Chemical Industry, Xinglong Construction, and White Carbon Black Fine Chemical Industry, constitute the Xinglong inorganic silicon chemical industry cluster. The industrial wastewater discharged by these chemical enterprises contains a large amount of Ni, Cu, Co, and some other trace elements, which enter groundwater through precipitation and runoff, thus increasing trace element content in groundwater^35^. Also, in the northwest region of the study area, the content of other trace elements, except Co and Ni, in the Weishui River basin is relatively higher. It is possible that these trace elements were from the mining and smelting activities and were transported with hydrologic gradients^36^.

The spatial variation of both carcinogenic substance (Cr) and non-carcinogenic substance (Ba) was less than 100%, that is 50.57% and 28.22%, respectively. Relatively low variation implied Cr and Ba in groundwater were less influenced by human activities. It can also be seen from the spatial distribution map that the content of Cr and Ba was evenly distributed throughout the study area, indicating that the high content of these two trace elements may be caused by natural non-point source pollution. Generally, Ba in waters is primarily derived from natural sources of weathering and subsequent pedogenesis^37^. Though Cr in all the samples met the standard and was distributed evenly, several sampling sites had relatively high Cr concentrations, which were significantly greater than average value. This indicates the influence of localized human activities.

Pb, Mo, and Fe elements are distributed in a scattered pattern in space, and the areas with high content mainly include the Ma’an Village of Zhuzhou city, the Lishutang Village of Zhuzhou county, the Xinglongshan Village of Zhuzhou city, the Yunnong demonstration area of Ma’an Village of the Zhuzhou city, and the Shitan Town of the Xiangtan City, which may be related to the surrounding point source pollution. Sewage generated by chemical plants, automobile repair factories, and rural waste that is discharged directly into the river without treatment, leads to the increase of Pb, Mo, and Fe content in groundwater. This is consistent with findings of other researchers^15,38^ that Pb mainly comes from lead deposits, soot emissions from coal-burning, and agricultural inputs.

**Figure 4:**
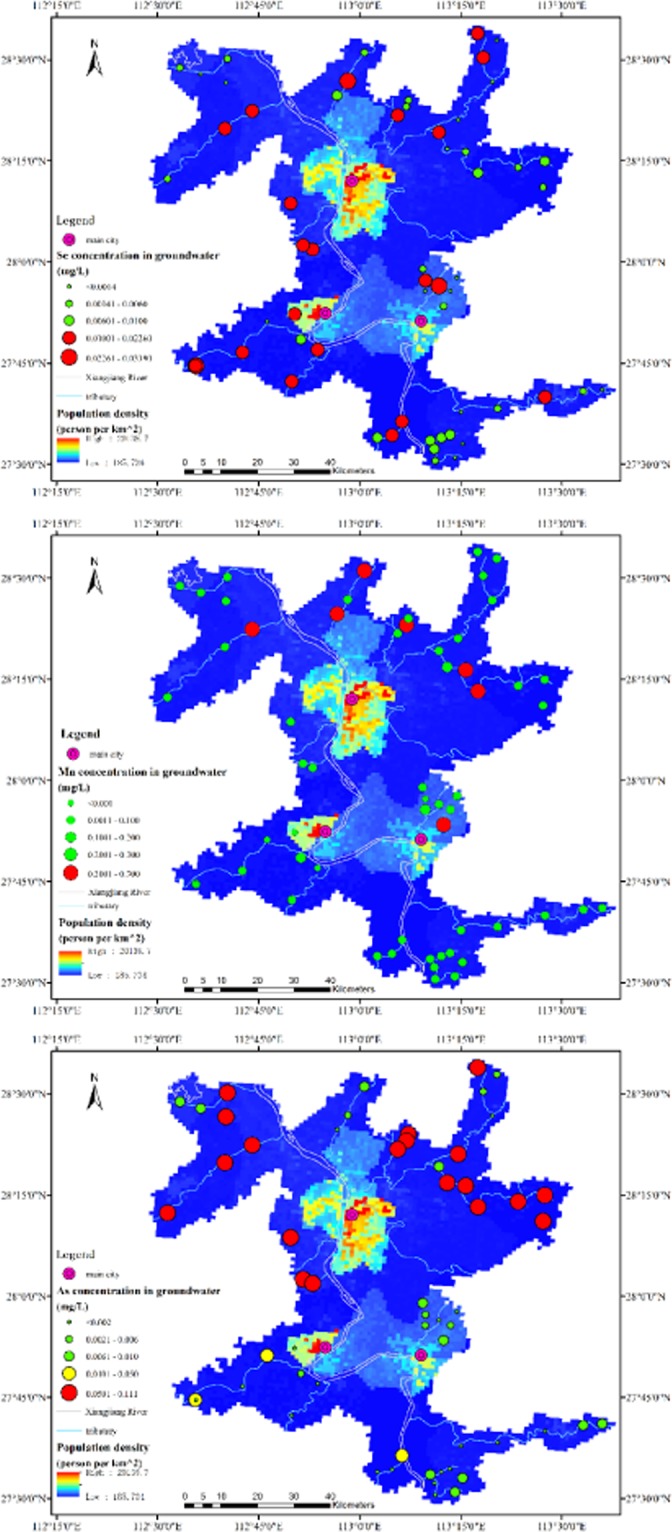
Spatial relationship between concentration of Se, Mn, and As against population density in study area. (ArcGIS Desktop. 10.3. ESRI, California, US. https://desktop.arcgis.com).

To understand the relationship among trace elements distribution, risk patterns, and population distribution, we draw the figures about the concentrations of Se, Mn and As in samples against population density (Fig. 4), due to their significant over-standard rate.

There were 37.93% of all samples with over-standard Se concentration. According to Fig. 4, most of those high concentration points were at the west side of Xiangjiang River, where has rich coal reserves and a long history of coal mining. Se is usually an associated mineral of coal, especially the lignite deposits. Other high concentration points of Se along the Laodao River might be a result of copper mining and smelting in the northeast of study area (Fig. 4). All these high concentration areas are not only threatening the health of people living in, but also posing risks to downstream big cities with high population density.

The sampling sites with over-standard Mn concentration were discretely distributed in the north of study area. Mn concentrations of other sites, however, were far lower than the over-standard ones. That might indicate small-scale human activities which usually would not pose risks to downstream.

Concentration of As in 32.8% of samples exceed the Chinese groundwater standard (0.05 mg/L). Most of these samples deriving from the sampling points located around Changsha city, along with the Laodao River in the northeast and Weishui River in the northwest (Fig. 4). Due to the special geological conditions of study area, high As concentrations in rock bed have significant influence on soil and adjacent groundwater. Considerable amount of As could be transferred from groundwater to tributaries by groundwater discharge and directly move into Xiangjiang River, which is the drinking water source for Changsha, a city with more than 8 million people dwelling in.

### Health risk assessment

Based on the health risk assessment model, the health risk and carcinogenic risk caused by trace elements through ingestion and dermal absorption of trace elements in groundwater in the core area of the Chang-Zhu-Tan urban agglomeration were calculated. In general, when HI is less than 1, the health risks to human are within acceptable risk levels. As shown in Table 3, HQ of non-carcinogenic substances (Ba, Cu, Fe, Mn, Mo, Ni, Pb, Se, and Zn) caused by drinking water and skin contact are all less than 1. The health risk values caused by 12 trace elements in groundwater are within the range of 1.64 × 10^−4^ to 1.25 × 10^−1^. HQ of Mn and Ba is the highest, while the HQ of Cu is the lowest. Çelebi *et al*.^21^ also demonstrated that higher HQ value of Mn was found in groundwater of Melen watershed, Turkey. Mn is a basic element of metabolism and is derived from foods for human consumption. Of all the target tissues, the brain is the most sensitive to Mn. Drinking Mn-rich water causes neurological diseases.

**Table 3:** Hazard quotients of all trace elements and cancer risks of Cd, Cr, and Pb in the study area.

Elements	RfD_ingestion_^a^(μg/kg/day)	RfD_dermal_^b^(μg/kg/day)	SF^c^(mg/kg/day)	HQ_ingestion_^d^	HQ_dermal_^e^	HQ^f^	CR^g^
Ba	70	14	—	1.23 × 10^−1^	1.11 × 10^−3^	1.25 × 10^−1^	
Cd	0.5	0.005	6.1	1.28 × 10^−3^	2.31 × 10^−4^	1.52 × 10^−3^	3.92 × 10^−6^
Co	0.3	0.0003	—	1.23 × 10^−2^	8.86 × 10^−3^	2.12 × 10^−2^	
Cr	3.0	0.015	0.5	3.34 × 10^−2^	2.40 × 10^−2^	5.74 × 10^−2^	5.00 × 10^−5^
Cu	40	12	—	1.63 × 10^−4^	9.79 × 10^−7^	1.64 × 10^−4^	
Fe	300	45	—	1.76 × 10^−3^	2.11 × 10^−5^	1.78 × 10^−3^	
Mn	20	0.8	—	1.20 × 10^−1^	5.41 × 10^−3^	1.25 × 10^−1^	
Mo	5	1.9	—	2.48 × 10^−3^	1.18 × 10^−5^	2.49 × 10^−3^	
Ni	20	5.4	—	1.99 × 10^−3^	2.66 × 10^−6^	2.00 × 10^−3^	
Pb	1.4	0.42	0.055	5.19 × 10^−2^	3.11 × 10^−4^	5.22 × 10^−2^	4.00 × 10^−6^
Se	5	2.2	—	5.26 × 10^−2^	2.15 × 10^−4^	5.28 × 10^−2^	
Zn	300	60	—	7.45 × 10^−3^	4.02 × 10^−5^	7.49 × 10^−3^	
HI^h^/TCR^i^				4.09 × 10^−1^	4.02 × 10^–2^	4.49 × 10^−1^	5.79 × 10^−5^

^a^Reference Dose (ingestion); ^b^Reference Dose (dermal); ^c^Slope Factor; ^d^Hazard Quotient (ingestion); ^e^Hazard Quotient (dermal); ^f^Hazard Quotient; ^g^Cancer Risk; ^h^Hazard Index; ^i^Total Cancer Risk.

Although drinking Mn-contaminated water has shown no carcinogenic or mutagenic effects, it can cause high blood pressure^39^. Therefore, Mn and Ba levels in groundwater need to be closely monitored.

For each of these trace elements, HQ ingestion is higher than HQ dermal, which means that the health risks of various non-carcinogenic substances caused by ingestion are higher than those caused by dermal absorption. In terms of the method of ingestion, the exposure risk of Ba element is the highest (1.23 × 10^−1^), while that of Cu element is the lowest (1.63 × 10^−4^). The sequence of health risk associated with are non-carcinogenic drinking water is, as follows: Cr > Co > Mn > Ba > Pb > Cd > Se > Zn > Fe > Mo > Ni > Cu. Cr has the highest health risk of 2.40 × 10^−2^ through skin contact, while Cu has the lowest exposure risk of only 9.79 × 10^−7^. The order of skin exposure risk of various trace elements is Cr > Co > Mn > Ba > Pb > Cd > Se > Zn > Fe > Mo > Ni > Cu. In addition to Cr, the health risk value of dermal absorption exposure was 1–3 times smaller than that of ingestion. In general, the health risk of 13 trace elements through ingestion and dermal absorption was less than 1. This indicates that in well waters in the core area of the Chang-Zhu-Tan urban agglomeration, the health risks caused by the trace elements of As, Ba, Cu, Fe, Mn, Mo, Ni, Pb, Se, and Zn through ingestion and dermal absorption are small, and they will not pose obvious harm to the exposed population.

In this study, the contents of carcinogens Cr, Pb, and Cd did not exceed the standard value of groundwater in China (Table 1). The TCR value is 5.79 × 10^−5^, which is within the acceptable risk level (10^−6^ ~ 10^−4^). Among the ways of ingestion, Cr was the highest (5.00 × 10^−5^), which is 12.5 times and 12.8 times of the carcinogenic risk of Pb and Cd, respectively. Hunan province ranks sixth in China in terms of Cr emissions. The Cr emission in the Chang-Zhu-Tan area accounts for about one-third of the total Cr emission in Hunan province^15^. Therefore, Cr appears to be the main pollutant source to produce cancer among these trace elements. Special attention should be given to the management of Cr pollution in the waters in the study area. So far, although China has formulated groundwater environmental quality standards, the evaluation model of health risk of carcinogens in water still needs to apply the evaluation model recommended by EPA, which is influenced by different regions, residents’ height and weight, drinking water intake rate, and life expectancy. Currently, there is no agreed limit for acceptable maximum carcinogenic and non-carcinogenic risk levels in China. Therefore, this study is only a preliminary assessment of the health risk of groundwater in the core area of the Chang-Zhu-Tan urban agglomeration. Future work should focus on these specific problems to improve the health risks of the water environment in China’s urban agglomeration construction.

It is important to note that the determination of trace element pollutants in our study of other elements through ingestion is far higher than that of dermal absorption, and the amount of Cr by ingestion and dermal absorption risk value is 3.34 × 10^−5^ and 2.40 × 10^−5^, respectively, and cancer risk value of Cr element is high. Therefore, the dynamic change of groundwater in the Cr should be monitored closely. Besides, all kinds of trace elements on human health hazards are not independent, but there is a certain inner link. While the interaction among various risk factors, environment, individual genes, and lifestyle together determines the level of health risks, there is no unified way to assess the health risks caused by various mixed risk factors. This will also be the topic of further research.

## Conclusion

The average concentration order of the 13 trace elements in groundwater in the core area of the Chang-Zhu-Tan urban agglomeration was Ba > Mn > Zn > Fe > Se > Cr > Pb > Ni > Mo > Cu > Co > Cd. The concentrations of Ba, Mn, Zn, and Fe were 0.28 mg L^−1^, 0.08 mg L^−1^, 0.07 mg L^−1^ and 0.02 mg L^−1^, respectively. The concentrations of the three carcinogenic substances, namely Cd, Cr, and Pb were 2.1 × 10^−5^mg L^−1^, 3.2 × 10^−3^ mg L^−1^, and 2.3 × 10^−3^ mg L^−1^, respectively. Compared with China’s groundwater environmental quality standard (GB/T 14848–9), the four elements of Se, Mn, Zn, and Ni have exceeded the standard, with the highest exceeding rate of 37.93%, followed by Mn (17.24%). A few samples of Zn and Ni have exceeded the standard, with an exceeding rate of 1.72%. None of the three substances exceeded the standard. Areas with high groundwater trace element content were mainly distributed in the upper reaches of the Xiang River, the Xinglongshan Village in Zhuzhou City, and the surrounding areas of mining areas.

Health risk assessment indicated that the health risk caused by non-carcinogenic substances through drinking water was higher than that caused by skin exposure. The HQ of non-carcinogenic substances (Ba, Cu, Fe, Mn, Mo, Ni, Pb, Se, and Zn) caused by drinking water and skin contact are all less than 1. Furthermore, the HI value was also less than 1, which was an acceptable risk level. However, Mn and Ba have higher health risk values than other trace elements. Among carcinogenic substances, Cr has the highest carcinogenic risk value, which is close to the maximum acceptable carcinogenic risk value. It is necessary to pay close attention to the dynamic changes of Cr, Mn, and Ba content in groundwater.

## Data Availability

All data generated and/or analyzed during this study are available from the corresponding author on reasonable request.
